# Distribution of CYP2D6 multiplication, CYP2D6*5, and clinical implications in postoperative patients receiving tramadol analgesia in the Minangkabau ethnic group, Indonesia

**DOI:** 10.12688/f1000research.155988.1

**Published:** 2024-12-17

**Authors:** Rinal Effendi, Aufa Rizqia Haz, Asrul Muhammad Fuad, Bagus Dermawan, Nuruliawaty Utami, Desriani

**Affiliations:** 1 Department of Anesthesiology, Faculty of Medicine, Universitas Andalas, Padang, West Sumatra, Indonesia; 2 Research Center for Genetic Engineering, Soekarno Sains and Technology Park, National Research and Innovation Agency (BRIN), Jl. Raya Bogor Km, 46 Cibinong, 16911, Indonesia; 3 Department of Neurology, Jl dr. Soetomo no 16, Dr Kariadi General Hospital Medical Center, Semarang, Central Java, 50244, Indonesia

**Keywords:** CYP2D6, Long PCR, Opioid Analgesic, Post Operative, Tramadol, West Sumatera

## Abstract

**Background:**

Cytochrome P450 2D6 (CYP2D6) is an important enzyme that metabolizes commonly used drugs, such as tramadol hydrochloride (a widely used opioid analgesic). Genetic polymorphisms of CYP2D6 have been shown to influence the pharmacodynamic properties of administered drugs. This study aimed to screen 63 postoperative patients (wild-type, CYP2D6*5, and CYP2D6 multiplication) of Minangkabau ethnicity in West Sumatera, Indonesia, who received tramadol using a modified long PCR method, and to investigate the clinical impact of tramadol on these patients. This study had a retrospective clinical trial registry number NCT06642480.

**Methods:**

The Reported Long PCR was modified using different DNA polymerases, optimizing annealing, and the number of step PCR to detect wild-type, CYP2D6*5, and multiplication CYP2D6. To ensure accurate PCR, the size of the PCR product was monitored: wild-type (5kb), CYP2D6*5 (6kb), and CYP2D6 multiplication genotype (10 kb). The wild-type PCR product was used as the internal control. The method was applied to screen 63 postoperative patients of Minangkabau ethnicity who received tramadol. The clinical impact was investigated and analyzed using analysis of variance (ANOVA) followed by a chi-square test.

**Result:**

The Long PCR was successfully modified with a two-step PCR at 68°C °C as the annealing and extension temperatures. The screened genotype patients were dominated by the wild-type, followed by CYP2D6*5, and multiplication CYP2D6 with percentages of 89%, 6,3%, and 4,7% respectively. A total of 6.3% of CYP2D6*5 cases were classified as heterozygous and predicted as intermediate metabolizers. In addition, sex and age did not affect postoperative tramadol analgesia treatment, whereas weight had a significant impact (p=0.008).

**Conclusion:**

The percentages of CYP2D6*5 and CYP2D6 multiplication genotypes were similar to those observed in another Asian population. Based on statistical analysis, tramadol was effective as an analgesic treatment for postoperative Minangkabau ethnicity patients with wild-type, CYP2D6*5, and multiple CYP2D6 genotypes. Further studies with larger sample sizes and longer follow-up periods are required to confirm these findings.

## 1. Introduction

Individuals vary in their P450 2D6 (CYP2D6) activity levels, ranging from complete lack of metabolism of certain drugs to ultra-rapid metabolism, which can lead to adverse effects during standard treatment. CYP2D6 metabolizes 15-25% of clinically used drugs, including a broad spectrum of medications such as antidepressants (paroxetine, tricyclic antidepressants, etc.), analgesics (codeine, tramadol, oxycodone, etc.), oncology (tamoxifen, etc.), antihypertensives (metoprolol, bisoprolol, etc.), and cardiology drugs. The gene encoding the enzyme is located on chromosome 22q13.1, and contains nine exons. It is positioned alongside two pseudogenes, CYP2D7 and CYP2D8, which are highly homologous to the transcribed active area with a similarity percentage of 94.2% and 89.1%, respectively. These two pseudogenes were also composed of l9 exons. ^1^ Based on metabolic capacity, the phenotype of CYP2D6 can be divided into four parts: poor metabolizer (PM), intermediate metabolizer (IM), extensive metabolizer (EM), and ultra-metabolizer (UM). ^2^ The PM or IM phenotype can have detrimental effects on patients because of their inability to effectively metabolize drugs. In contrast, patients with the UM phenotype may also be at risk of therapeutic failure or increased toxic side effects of metabolites. These phenotypic differences alter the capacity of enzymes to metabolize drugs. Phenotypic variations in CYP2D6 have been attributed to polymorphisms, deletions, and variations in the number of copies of the enzyme. The Pharmacogene Variation (PharmVar) Consortium classified the gene as highly polymorphic. Further details can be found at https://www.pharmvar.org/gene/CYP2D6. ^3^

The frequency of CYP2D6 alleles, especially CYP2D6*5, varies worldwide. According to a previous study, the frequencies of CYP2D6 in Hong Kong were UM 3.3%, 49.9%, 46.4%, and 0.4%. ^4^ CYP2D6*5 associated loss of function allele varied by 3-6% in African, European, and East Asian populations. ^5^ CYP2D6*5 heterozygotes with the IM phenotype were 2.4% in Malay, 2.2% in Indian, and 0.5% in Chinese, while the PM phenotype was 11% for Chinese, 5% for Indian and 4.8% for Malay ethnicity in the Singapore population. ^6^

Tramadol hydrochloride is a popular analgesic opioid that is widely used for the treatment of postoperative, dental, cancer, neuropathic, acute musculoskeletal neuropathic, and acute musculoskeletal pain. It is associated with high clinical efficacy, a low incidence of adverse effects, and low abuse potential. Tramadol can be administered in several ways, including oral, rectal, sustained-release, and parenteral (IV/IM). Tramadol is converted into two active metabolites: M1 (O-desmethyltramadol) and M2 (N-desmethyltramadol). CYP P450 2D6 catalyzes o-demethylation to M1 (the main analgesic effective metabolite), while CYP2B6 and CYP3A4 catalyze n-demethylation to M2. The main route of elimination of tramadol and its metabolites is through the kidneys. The average elimination half-life is approximately 6 h. ^7, 8^ The typical dosage of tramadol administered was within the range of 50–100 milligrams. ^9^ The dosage for pediatric patients was 50 mg, whereas the adult dosage was 100 mg. As mentioned above, CYP2D6 is polymorphic, with different alleles encoding functionally different enzymes. This suggests that different genetics would influence the pharmacokinetics and/or pharmacodynamics of tramadol. ^7, 8, 10, 11^

West Sumatra is a province in Indonesia, and its population comprises the Minangkabau ethnic group. Previous studies have reported the analgesic effect of endogenous opioids and receptor polymorphisms of OPRM A118G and COMT G158A in the Minangkabau ethnic group. The results showed that gene polymorphisms were not significantly associated with pain sensitivity, and there is still limited information on gene polymorphisms related to opioid analgesics in West Sumatra. ^1^

CYP2D6 genetic detection technologies have been developed, including TaqMan technology, microarray, Southern blotting, PCR-RFLP, Long PCR, and single-strand conformation polymorphism (SSCP). ^12– 15^ The methods used to count the number of CYP2D6 copies were Southern blotting, TaqMan technology, HRM qPCR, ^16, 17^ and pyrosequencing genotyping. ^18^ Johansson et al. (1995) reported a method for detecting multiplication and deletion using Long PCR was reported by Johansson et al. 1995. ^19^ This method used rTth DNA Polymerase from Perkin Elmar and 0.09U Vent Polymerase (New England Biolabs) in a total volume of 25 μL. PCR products with size of 10kb, 6kb, and 5kb signify multiplication, CYP2D6*5, and wild-type. ^19^ This study modified the long PCR method reported by Johansson et al. (1994) to screen for multiplication, CYP2D6*5, and wild-type CYP2D6 in post-operative Minangkabau patients receiving tramadol analgesia. The genetic distribution and clinical effects of tramadol were also investigated.

## 2. Methods

### 2.1 Determination of Patients' Visual Analogue Scale (VAS)

“The study was approved on 21 March 2022, by the Ethics Committee of the Faculty of Medicine, University of Andalas West Sumatra, Indonesia (number 653/UN.16.2/KEP-FK/2022). Informed consent was also signed and obtained from the patients for blood collection, relevant clinic data collection, and determination of the patients' visual analog scale (VAS). These studies have fulfilled WMA declaration of Helsinki-Ethical principles … In addition, this study had a retrospective clinical trial registry number NCT06642480. It is our first time using the clinical trial registry because the research already finished, so we applied for a retrospective registry clinical trial. All clinical trials were registered, allowing information to be shared between clinicians, researchers, and patients. This increases public confidence in the research. A clinical trial registry (or retrospective clinical trial registry) can be enrolled and issued at https://register.clinicaltrials.gov/.

The VAS scores of 63 patients were determined by injecting tramadol at a dose of 100 mg 30 min before the operation was completed. This was observed in the recovery room at 30, 60, and 120 min. Tramadol had an onset time of 15-60 minutes, with peak effectiveness at 2-6 hours postoperatively. Pain level was categorized as 0 (no pain), 1-3 (signifying mild), 4-6 (middle), and 7-10 (heavy pain).

### 2.2 Blood sample collection and DNA extraction

Blood samples from 63 postoperative patients receiving tramadol analgesia and another two samples were collected and stored in a tube containing ethylenediaminetetraacetic acid. Subsequently, DNA was extracted using the Purelink Genomic DNA Mini Kit from Thermo Fisher Scientific (# K1820-01). The extracted DNA was further verified using 1% agarose, Ist Base #BIO-1000-500g and its concentration was measured using a spectrophotometer at A260/280.

### 2.3 Long PCR modification for deletion, multiplication, and wild-type CYP2D6 genotyping

The long PCR method of Johansson et al. (1994) was modified using Promega GoTaq Long PCR Master Mix #M4021, with the following DNA primers: Lx2F: 5′ GCC ACC ATG GTG TCT TTG CTT TC 3′, Lx2R: 5′ ACC GGA TTC CAG CTG GGA AAT G 3′ for multiplication detection amplification, DF: 5′ GCC ACT CTC GTG TCG TCA GCT TT 3′; DR: 5′ GGC ATG AGC TAA GGC ACC 3′ for detecting CYP2D6*5 deletion, LongPCR-CYP2D6-Fw: 5′ CCA GAA GGC TTT GCA GGC TTC A 3′, and LongPCR-CYP2D6-Rev: 5′ ACT GAG CCC TGG GAG GTA GGT A 3′ for wild-type detection amplification and serving the purpose of internal control. Successful PCR optimization of DNA amplification resulted in 10 kb, 6 kb, and 5 kb fragments for multiplication of the CYP2D6 gene, CYP2D6*5, and wild-type, respectively. Three tubes were required to genotype each patient sample. Each tube contained the DNA primers described above. Two samples with pain level scales of 0 and 8 were selected as DNA templates for Long PCR modifications. DNA samples were selected based on VAS criteria. The Long PCR ingredient of each amplification target contained 25μl GoTaq ^®^ Long PCR Master Mix Promega #M4021, 1 μl FW primer 10 μM, 1 μl Rev primer 10 μM, 1 μl DNA template, and 22 μl nuclease-free water. Each PCR amplification included a Negative Control (NTC) containing the same ingredients, except that the DNA template was substituted with nuclease-free water. The two-step PCR was optimized using the gradient PCR method. Amplification was performed using a Kyratec SuperCycler. SC200 (Kyratec Life Science) The conditions included pre-denaturation at 95°C for 2 min, followed by 30x amplification with denaturation at 94°C for 30 sec, and annealing temperature of 63/63,95/65,2/67,15/68°C for 12 min, followed by post-extension at 72°C for 10 min.

### 2.4 Statistical analysis

The demographic characteristic data were analyzed using analysis of variance (ANOVA), followed by a chi-square test. Statistical analysis was conducted using IBM SPSS software version 24, with p values less than 0.05. related as statistically significant.

## 3. Result

### 3.1 Long PCR modified from Johannson *et al.* ^19^

**Figure 1.**
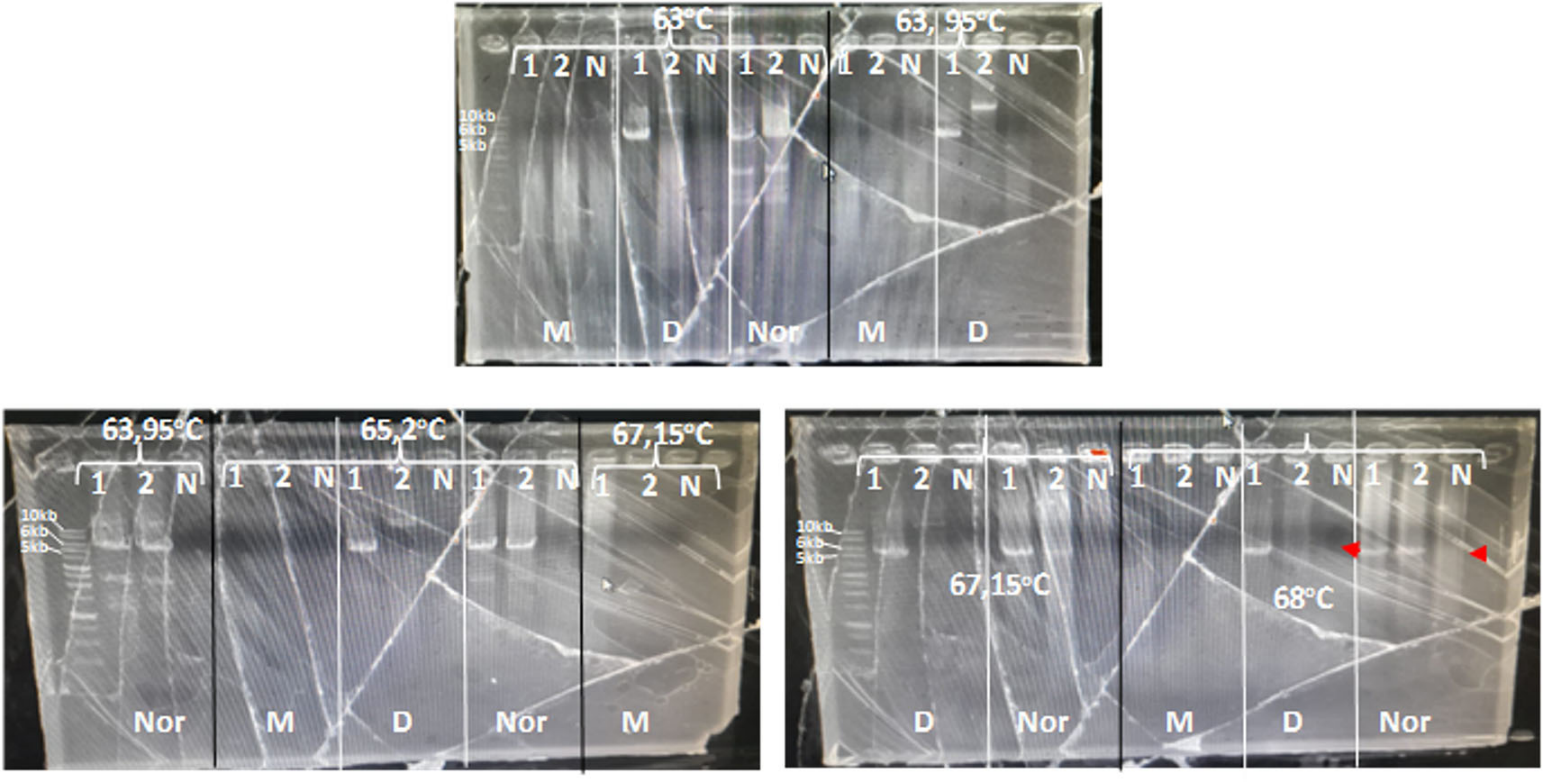
Long PCR annealing temperature optimization. Sample: 1, 2. N: No template control. M: for multiplication detection, D: for CYP2D6*5 detection, and Nor: for Wild-type detection; 10 kb, 6 kb, and 5 kb for multiplication, CYP2D6*5, and wild-type CYP2D6.

Long PCR was selected for CYP2D6*5 or multiplication and detection of wild-type CYP2D6. Although this method is time-consuming, it is more cost-effective than the other methods. The long PCR method developed by Johanson et al.(1994) ^19^ was modified in this study. The PCR products of wild-type CYP2D6, CYP2D6*5, and multiplication detection yielded 5 kb, 6 kb, and 10 kb, respectively. In contrast to Johanson et al. (1994), who used PerkinElmer's rTDNA and New England Biolabs' 0.09U Vent, the present study used Promega's GoTaqLong PCR Mastermix #M4021. GoTaq ^®^ Long PCR Master Mix uses a combination of recombinant hot-start Taq DNA polymerase and recombinant proofreading DNA polymerase. The amplification conditions must be further optimized. In a preliminary study, the annealing temperature of 55°C-67°C was optimized using a three-step PCR (denaturation, annealing, and extension) for three DNA primers. No PCR product was detected (data not shown). Further, the annealing temperature across five different temperatures starting from 65 °C to 68°C was optimized using two-step PCR (denaturation, annealing also as an extension step) for each DNA primer. Among the five annealing temperatures tested, 68°C was the best temperature. One patient DNA sample with an 8 VAS scale, produced 6kb and 5kb for the amplified CYP2D6*5 DNA primer and wild-type CYP2D6 DNA primer, respectively, with no PCR product in all NTC. DNA samples from other patients that had a VAS score of 0 shown only produced 5kb for the wild-type DNA primer. In addition, the DNA primer used for the detection of CYP2D6 amplification did not generate a PCR product for either sample ( Figure 1). This showed that the two samples used for optimization were CYP2D6*5 and the wild-type. Specifically, CYP2D6*5 was identified as heterozygous CYP2D6*5 as the PCR product was produced using both CYP2D6*5 and wild-type DNA primers. According to Johansson *et al.,* ^19^ heterozygous CYP2D6*5 led to PCR products with both CYP2D6*5 and wild-type DNA primers, whereas homozygous CYP2D6*5 resulted in no PCR product with wild-type DNA primers.

**Figure 2.**
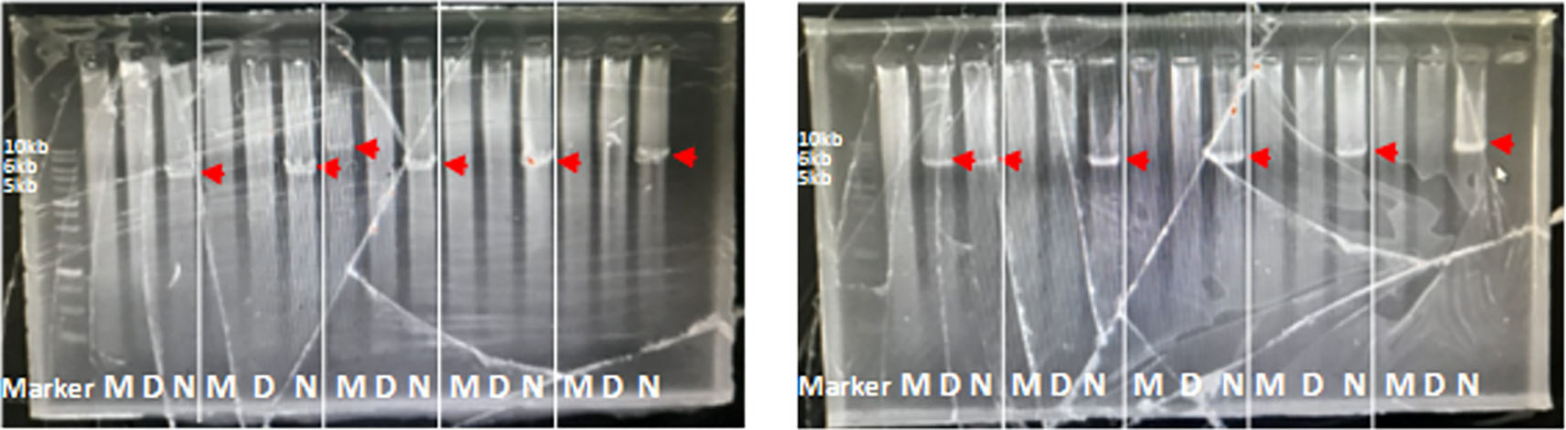
Long PCR screening patient sample for detection of CYP2D6*5, multiplication and wild-type. The target was shown with the red arrow: 10 kb, 6 kb, and 5 kb for multiplication, CYP2D6*5, and wild-type CYP2D6.

**Figure 3.**
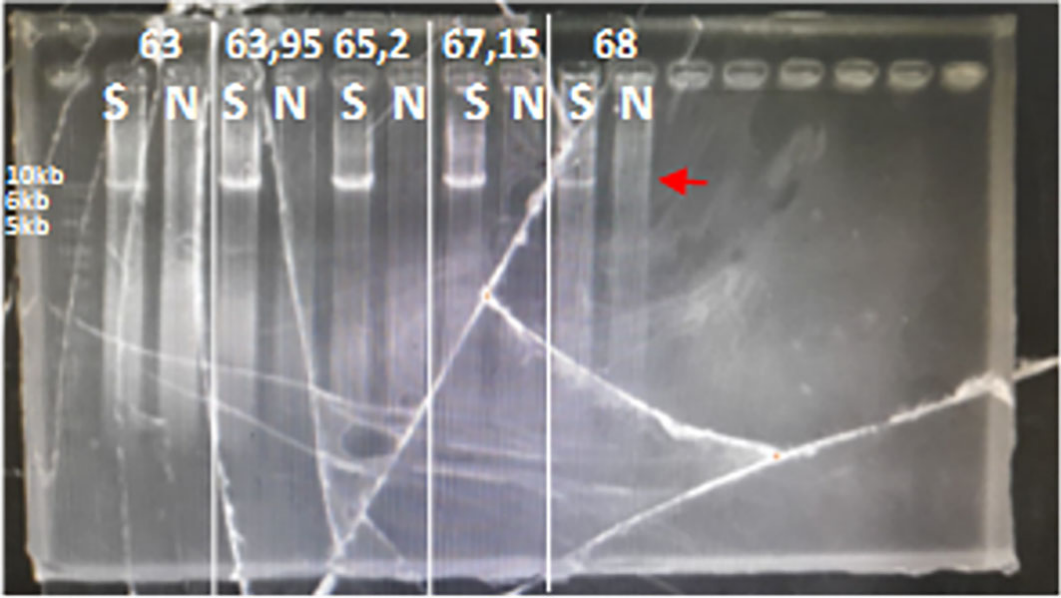
Long PCR annealing PCR optimization for multiplication CYP2D6. The target was shown with the red arrow, 10 kb.

The optimal PCR setup was used to screen several samples. The screening results showed that one sample produced 10kb which was suspected to be a multiplication sample ( Figure 2). This sample was optimized for its optimal PCR setup, and the process was used to confirm and verify the results. Consistency was observed with a 10 kb PCR product as a single band across temperatures ranging from 63 to 68°C. The optimal PCR annealing temperature was determined to be 63.95, 65.2, and 67.15, indicating thick single-band DNA ( Figure 3). However, 68°C was selected because it produced a single band and was the most effective temperature for detecting both CYP2D6*5 and wild-type alleles. The remaining samples were screened using a modified method.

### 3.2 Distribution of CYP2D6*5, or multiplication, and wild-type genotype of Minangkabau ethnicity

**Figure 4.**
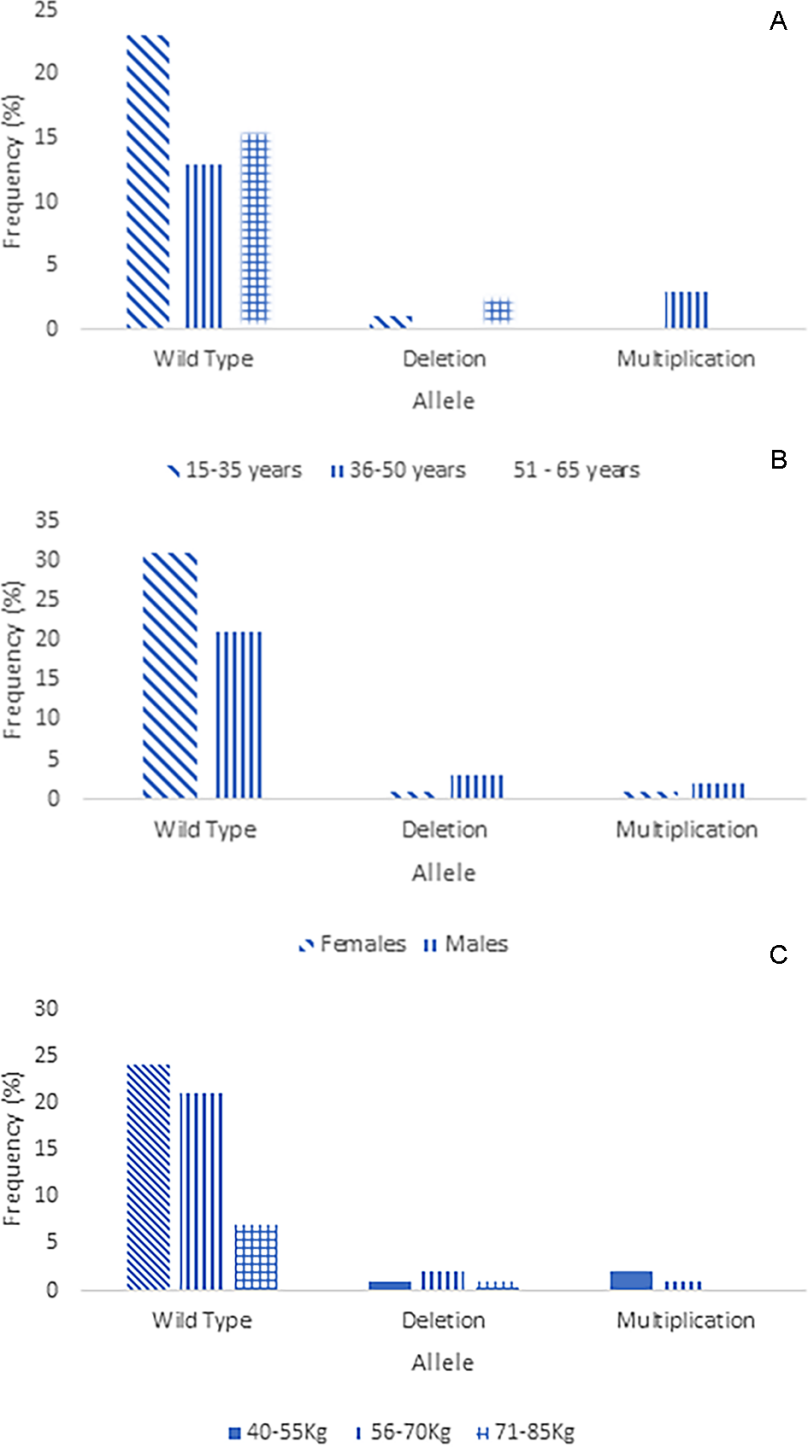
Distribution of multiplication, CYP2D6*5 and wild-type CYP2D6 in Minangkabau ethnicity. A. Based on age, B. gender, and C. weight.

As previously reported, the modified Long PCR was applied to postoperative patients of the Minangkabau ethnicity who were administered tramadol as an analgesic. 63 patients who received tramadol analgesia were tested for genetics, while the other two samples did not undergo genetic testing because they had no detailed information. Out of 63 DNA patient samples, four samples could not be amplified due to poor DNA quality. The distribution of patients with multiplication, wild type, or CYP2D6*5 based on age, sex, and weight is shown in the following graphic ( Figure 4). The majority of patients of Minangkabau ethnicity had a wild-type genotype (89%). The frequencies of CYP2D6*5 and multiplication were 6.3% and 4.7%, respectively, for each genotype. CYP2D6*5 is a heterozygous allele, as all samples produced 6 and 5 kb PCR DNA products in tubes containing CYP2D6*5 and wild-type DNA primers, respectively. The distribution of CYP2D6*5 and the multiplication genotypes in some areas are shown in Table 1.

**Table 1.** Comparison of CYP2D6*5, multiplication genotype distribution in some areas.

Ethnic groups	Genotype (%)		Ref.
	CYP2D6*5 (Deletion)	Multiplication	
Eastern Han Chinese	4.82	0.69	26
Central Han Chinese	7.17	1.35	26
Malaysia Chinese	2.54	4.24	26
Malay	2.4	4.8	6
Asia	6.24	Not reported	20
Japan	6.3	4.6	16
Minangkabau	6.3	4.7	Present report

The modified Long PCR method is straightforward and highly accurate for genotyping. The method was highly reproducible and accurately reproduced the PCR product sizes reported by Johansoon et al. (1996 for each genotype. ^19^ Reproducibility was used as a parameter to control the precision of PCR.

### 3.3 Patients demographics

The administration of tramadol (100 mg) to the patients resulted in optimal pain relief. This was evidenced in the present study, which showed a significant association between the drug and weight of postoperative patients (p = 0.008). In addition, wild-type genotypes, heterozygote CYP2D6*5, and multiplication were observed in all the patients. Age and sex showed no significant correlations (p = 0.052 and 0.243, respectively) ( Table 2). Furthermore, the VAS scores were in 50% to 60% agreement with molecular detection. Table 2 shows that there was no correlation between the VAS score and molecular detection. The VAS is a subjective measure that depends on the pain tolerance of patients and the type of surgery. However, recent studies have not differentiated between the various classifications of surgery.

**Table 2.** The characteristic demographic of deletion, multiplication and wild-type polymorphism of CYP2D6 (age, sex, and weight as VAS measurement).

Characteristics	Distribution	Molecular screening				Total (n)	p-value
		EM (Wild-type)	IM (CYP2D6*5 Heterozygot)	UM (Multiplication)	No details		
Age	15-35 years	23 (85.2%)	1 (3.7%)	0 (0.0%)	3 (11.1%)	27	0.052
	36-50 years	13 (76.5%)	0 (0.0%)	3 (17.6%)	1 (5.9%)	17	
	51-65 years	16 (76.2%)	3 (14.3%)	0 (0.0%)	2 (9.5%)	21	
Gender	Female	31 (81.6%)	1 (2.6%)	1 (2.6%)	5 (13.2%)	38	0.243
	Male	21 (77.8%)	3 (11.1%)	2 (7.4%)	1 (3.7%)	27	
Weight	40-55 kg	24 (82.8%)	1 (3.4%)	2 (6.9%)	2 (6.9%)	29	0.008
	56-70 kg	21 (80.8%)	2 (7.7%)	1 (3.8%)	2 (7.7%)	26	
	71-85 kg	7 (87.5%)	1 (12.5%)	0 (0.0%)	0 (0.0%)	8	
	No information	0 (0.0%)	0 (0.0%)	0 (0.0%)	2 (100.0%)	2	
VAS value	0 (no pain)	13 (76.5%)	32 (11.8%)	1 (5.9%)	1 (5.9%)	17	0.244
	1-3 (signifying mild)	17 (85.0%)	1 (5.0%)	1 (5.0%)	1 (5.0%)	20	
	4-6 (middle)	5 (83.3%)	0 (0.0%)	0 (0.0%)	1 (16.7%)	6	
	7-10 (heavy pain)	16 (84.2%)	1 (5.3%)	1 (5.3%)	1 (5.3%)	19	
	No information	1 (33.3%)	0 (0.0%)	0 (0.0%)	2 (66.7%)	3	

## 4. Discussion

This study aimed to investigate CYP2D6*5 and CYP2D6 multiplication in post-operative Minangkabau ethnicity patients receiving tramadol analgesia and further investigate the efficacy of tramadol in these patients. To achieve this, the Long PCR method developed by Johansson *et al*., ^19^ was modified. The Long PCR, reported by Johansson *et al* 1994, was successfully modified with the GoTaq Long PCR master mix from Promega #M4021, with two-step PCR at 68°C annealing and extension PCR amplification temperatures. GoTaq ^®^ Long PCR Master Mix uses a combination of recombinant hot-start Taq DNA polymerase and recombinant proofreading DNA polymerase. Both enzymes in the GoTaq Long PCR master mix from Promega #M4021 were required to amplify long targets. Taq DNA Polymerase alone cannot correct nucleotide mismatches resulting from misincorporation and is therefore ineffective in amplifying segments longer than 3-5 kb. Products are truncated if nucleotides are misincorporated; further, they cannot be extended in subsequent cycles. Long amplicons can be amplified with a high yield by adding a small amount of proofreading enzyme to fix mismatches and allow extension. To ensure accurate PCR, the size of the product was monitored. The sizes of the wild-type, CYP2D6*5, and CYP2D6 multiplication PCR products were 5kb, 6kb, and 10 kb, respectively. The wild-type genotype PCR product was used as an internal control. Furthermore, heterozygous CYP2D6*5 produced a DNA PCR product in a tube containing CYP2D6*5 and wild-type DNA primers. In contrast, homozygous CYP2D6*5 produced no PCR products in the tube containing wild-type DNA primers. Although the real-time method offers a simpler and faster alternative to long PCR, it requires costly real-time PCR machines, and pyrosequencing is also costly. The modified Long PCR method was used to generate a distribution report of the Minangkabau ethnicity genotypes.

The majority of CYP2D6 genotypes were dominated by the wild-type genotype, with only 6.3% and 4.7% for CYP2D6*5 and multiplication, respectively. Furthermore, 6.3% of CYP2D6*5 patients were classified as heterozygous and intermediate metabolizers. These percentages are similar to the frequencies observed in other Asian populations for CYP2D6*5 and multiplication ( Table 1). Allele frequencies of CYP2D6 5* were 3.08% (European), 5.67% (African), 2.96% (Hispanic), 6.24% (Asian), and 1.30% (Samoan) respectively. ^20^ In Singaporean populations, ultra metabolizers presented 11, 5, and 4.8% of Chinese, Indian, and Malay ethnicity participants, CYP2D6*5 heterozygotes with IM phenotype were 2.4% for Malay, 2.2% for Indian, and 0.5% for Chinese ethnicity participants. ^6^

Deletion and multiplication of genes are important for phenotype prediction. Deletion of the entire CYP2D6 gene (*5) results in loss (in the homozygote) or reduction (in the heterozygote) of its protein production. ^2, 3, 21, 22^ It is important to acknowledge that drug metabolism in the multiplication genotype increases the activity of the enzyme at a high rate. ^3, 20^ This study showed that tramadol was significantly associated with the weight of postoperative patients receiving analgesia (p = 0.008). This indicated that administration of 100 mg of the drug resulted in optimal pain relief. As stated above, the typical dosage of tramadol administered is within the range of 50 to 100 milligrams. ^9^ The dosage for pediatric patients was 50 mg, whereas the adult dosage was 100 mg. Age and sex showed no significant correlations (p = 0.052 and 0.243, respectively). This could be interpreted as meaning that all genotypes in this activity individually metabolize the given tramadol is still possible. This also shows that both genotypes (heterozygote CYP2D6 *5 and multiplication) are clinically functional.

The potency of tramadol analgesia is approximately 10% that of morphine when administered intravenously. Tramadol has also been reported to be comparable to morphine. ^23, 24^

Tramadol is an efficient and well-tolerated medication for the treatment of chronic pain, whether of malignant or non-malignant origin, especially neuropathic pain. It can also be used to relieve pain related to trauma, renal or biliary colic, and labor. When taken as a non-opioid analgesic, the analgesic effect of the drug can be enhanced. ^23^ Furthermore, tramadol administration is ideally based on body weight. According to Sidiq *et al* (2015), 2 mg/kg body weight of tramadol hydrochloride is an optimal dose for postoperative analgesia when administered epidurally in urological surgery, with no significant increase in side effects. ^25^ Pang *et al* (2005) reported that the recommended intraoperative dose of tramadol is 2.5 mg/kg body weight for effective postoperative analgesia with minimal sedation, when considering the efficacy and side-effect profile. ^24^

Patients without CYP2D6 activity may not be able to metabolize drugs. Ultra-rapid metabolizers may also experience treatment failure owing to the rapid metabolism of active drugs, resulting in drug levels below the therapeutic range. ^3^ The data obtained specifically in the multiplication part were not in line with the presented statement. This discrepancy may be attributed to the absence of a subsequent follow-up. To enhance the quality of future data, it is essential to categorize the type of operation, collect a larger number of samples, and conduct longer follow-up periods.

## 5. Conclusion

In conclusion, the modified long PCR method for CYP2D6 genotyping successfully detected CYP2D6*5, multiplication, and wild-type genotypes. The proposed method is simple and highly accurate. To ensure accurate results, the size of the PCR products was monitored. The sizes of the wild-type, CYP2D6*5, and multiplication genotype PCR products were 5 kb, 6 kb, and 10 kb, respectively. The wild-type PCR product was used as an internal control. Furthermore, the majority of the CYP2D6 genotypes were dominated by the wild-type genotype, with only 6.3% and 4.7% for CYP2D6*5 and multiplication, respectively. The administration of tramadol (100 mg) to patients led to optimal pain relief, as evidenced by a study analyzed using IBM SPPS 24. The results showed a significant correlation (p = 0.008) with the weight of postoperative patients receiving tramadol analgesia but not with sex or age. This also suggests that in the Minangkabau ethnic group, all genotypes were capable of metabolizing the administered tramadol.

### Ethics and consent

The study was approved by the Ethics Committee of the Faculty of Medicine, University of Andalas West Sumatra, Indonesia (number 653/UN.16.2/KEP-FK/2022) on 21 March 2022. Informed consent was also signed and obtained from the patients for blood collection and determination of the patients' Visual Analog Scale (VAS). The participants in this study were 65 post-operative patients from M Djamil Central Hospital of West Sumatra and several hospitals near the capital of West Sumatra. Among the patients, 63 received tramadol analgesia and were tested for genetics, while the other two samples did not have detailed information. In the case of participants who were minors, consent was obtained and signed by their legal guardian or parent.

## Author roles

Desriani: Conceptualization, Data curation, Formal Analysis, Investigation, Project Administration, Funding Acquisition, Methodology, Validation, Writing-Original Draft Preparation, Writing-Review and Editing, Supervision; **Rinal Effendi:** Conceptualization, Funding Acquisition, Data curation, Formal Analysis, Investigation, Project Administration, Methodology, Validation, Writing-Original Draft Preparation, Writing-Review and Editing, Supervision; **Aufa RH**: Data Curation, Methodology, Investigation, Formal Analysis, Validation, Writing-Review and Editing; **Asrul MF**: Methodology, Formal Analysis, Validation, Writing-Review and Editing, Supervision; **Bagus Dermawan**: Methodology, Validation, Writing-Original Draft Preparation, Writing-Review and Editing, Supervision: **Nuruliawaty** Utami: Data Curation, Methodology, investigation, Formal Analysis, Validation, Writing-Review and Editing, Supervision.

## Data Availability

figshare: Distribution of CYP2D6 multiplication, CYP2D6*5, and clinical implications in postoperative patients receiving tramadol analgesia in the Minangkabau ethnic group, Indonesia https://doi.org/10.6084/m9.figshare.26870995. The project contains the following data:
• Dataset from 65 postoperative Minangkabau ethnic patients Figshare: Desriani, Desriani; Effendi, Rinal; Utami, Nuruliawaty; Dermawan, Bagus; Muhamad Fuad, Asrul; Rizqia Haz, Aufa (2024). Statistic analysis West Sumatera Patients.pdf. figshare. Dataset. https://doi.org/10.6084/m9.figshare.27895956.v1 The project contains the following data:
• Statistic analysis West Sumatera Patients.pdf Data is available under CC0 license.
