# Supplementary material for: Distribution of CYP2D6 multiplication, CYP2D6*5, and clinical implications in postoperative patients receiving tramadol analgesia in the Minangkabau ethnic group, Indonesia
Source: F1000Res. 2025 Sep 23;13:1526. Originally published 2024 Dec 17. [Version 3] doi: 10.12688/f1000research.155988.3 (PMC12508793; doi:10.12688/f1000research.155988.3)
Supplement: Peer review history

# Reviewer report: Reviewer response for version 3

**DOI:** 10.5256/f1000research.188479.r416788

**Published:** 2025-10-07

**Authors:** Heri Setiawan

**Affiliations:** 1 Universitas Indonesia, Depok, Indonesia

The manuscript has been revised, and all my concerns have been addressed adequately.

Is the work clearly and accurately presented and does it cite the current literature?

Partly

If applicable, is the statistical analysis and its interpretation appropriate?

Partly

Are all the source data underlying the results available to ensure full reproducibility?

Partly

Is the study design appropriate and is the work technically sound?

Partly

Are the conclusions drawn adequately supported by the results?

Partly

Are sufficient details of methods and analysis provided to allow replication by others?

Partly

Reviewer Expertise:

Pharmacology and toxicology

I confirm that I have read this submission and believe that I have an appropriate level of expertise to confirm that it is of an acceptable scientific standard.

# Reviewer report: Reviewer response for version 2

**DOI:** 10.5256/f1000research.185264.r407882

**Published:** 2025-09-16

**Authors:** Heri Setiawan

**Affiliations:** 1 Universitas Indonesia, Depok, Indonesia

Only one out of the six points of review has been adequately addressed, which is about the explanation of a normal metabolizer. Please revise and explain adequately each point, especially the association between the VAS value and the CYP2D6 polymorphism. However, the data showed significant different proportion (p = 0.008) between body weight (not VAS value) and CYP2D6 polymorphism.

Is the work clearly and accurately presented and does it cite the current literature?

Partly

If applicable, is the statistical analysis and its interpretation appropriate?

Partly

Are all the source data underlying the results available to ensure full reproducibility?

Partly

Is the study design appropriate and is the work technically sound?

Partly

Are the conclusions drawn adequately supported by the results?

Partly

Are sufficient details of methods and analysis provided to allow replication by others?

Partly

Reviewer Expertise:

Pharmacology and toxicology

I confirm that I have read this submission and believe that I have an appropriate level of expertise to confirm that it is of an acceptable scientific standard, however I have significant reservations, as outlined above.

# Response

**Published:** 2025-09-19

**Authors:** Desriani Desriani

**Affiliations:** Genetic Engineering, Not Applicable, Bogor, Jawa Barat, Indonesia

1. Only one out of the six points of review has been adequately addressed, which is about the explanation of a normal metabolizer. Please revise and explain adequately each point, especially the association between the VAS value and the CYP2D6 polymorphism. However, the data showed significant different proportion (p = 0.008) between body weight (not VAS value) and CYP2D6 polymorphism.

 > Thank you very much for your valuable input and comment. Please find below our explanation, which we have also included in the revised manuscript (in the abstract, methods, results, and discussion parts).

 In the present study, we used two different statistical methods:

1. Analysis of variance (ANOVA) was conducted to analyze the relationship between the amount of tramadol administered and the VAS value, as well as between the amount of tramadol administered and the molecular screening result.
2. The demographic characteristics data were analyzed with a Chi-Square test.

The statistical analysis was conducted with the IBM SPSS software version 24, with p values less than 0.05 being related as statistically significant.”

 The ANOVA analysis showed that administered tramadol was significantly associated with the VAS value of postoperative patients receiving analgesia with p = 0.045 (Supplementary). This result supported and indicated that administration of 100 mg of the drug resulted in optimal pain relief. In addition, the typical dosage of tramadol administered is within the range of 50 to 100 milligrams. ^10^ The dosage for pediatric patients was 50 mg, whereas the adult dosage was 100 mg. Furthermore, a significant association was also shown between tramadol administration and molecular screening, with p = 0.003 (in the Supplementary). This could be interpreted as meaning that all genotypes in this activity individually metabolize the given tramadol is still possible. This also shows that both genotypes (heterozygote CYP2D6 *5 and the multiplication) are clinically functional.

 In Table 2, the Chi-square test showed that the distribution of the molecular screening among the demographic factors (Age, Gender, and VAS value) of the West Sumatra patients does not have a significant correlation. While the weight of the patients is indicated as biased. As it showed inconsistent p-values between Pearson Chi-Square (p = 0.008), Likelihood Ratio (p = 0.149) and Linear-by-Linear Association (p = 0.124). According to McHugh 2013, too small sample size will affect to Pearson Chi Square, Likehood Ratio and Linear-by-linear P Value in the Chi-square test. Therefore, for a better understanding of this, further study needs a larger number of samples. In addition, gene polymorphisms are the most common type of genetic variation, defined as variations in gene sequences caused by changes in the DNA sequence that are heritable. ^29 ^Further, the VAS value has a high subjectivity, and open possibilities have no significant correlation with molecular screening, as proved by the Chi-square result (Table 2).

 2. "This study had a retrospective clinical trial registry number NCT06642480." ****In my opinion, it is not necessary to include the trial registry number in the abstract. "It is our first time using the clinical trial registry because the research already finished, so we applied for a retrospective registry clinical trial". This information may not be of concern to the reader

- Thank you very much for your valuable input and comment. We delete the sentences.

3. The images of the PCR product in gel electrophoresis are not clear

- Thank you very much for your valuable comment.  We are very sorry that our trays for visualizing PCR products were cracked when we conducted this research in 2022. This explains why the background of our PCR product figure was cracked. But according to us, the images are still readable and analyzable. Furthermore, in Figure 1, since M-D-Wt were not always in the same gel, we used a white line to indicate the same temperature annealing and a black line to indicate different annealing temperatures. This makes the data clearer and easier to read and analyze. In addition, We completed the figure label in Figure 1 (1-3), and also in Figure 2 (2-1 and 2-2).

There is an inconsistency about the patient number.

- Thank you very much for your valuable input and comment. We revised the sentences. A total of 65 patients participated in this study.  For detailed information, please kindly see in https://doi.org/10.6084/m9.figshare.26870995, as mentioned in the data availability section of the manuscript.

# Reviewer report: Reviewer response for version 2

**DOI:** 10.5256/f1000research.185264.r407883

**Published:** 2025-09-05

**Authors:** Anom Bowolaksono

**Affiliations:** 1 Cellular and Molecular Mechanisms in Biological System (CEMBIOS) Research Group, Department of Biology, Faculty of Mathematics and Natural Sciences, Universitas Indonesia, Depok, Indonesia

Most of comments are resolved

Is the work clearly and accurately presented and does it cite the current literature?

Yes

If applicable, is the statistical analysis and its interpretation appropriate?

Yes

Are all the source data underlying the results available to ensure full reproducibility?

Partly

Is the study design appropriate and is the work technically sound?

Partly

Are the conclusions drawn adequately supported by the results?

Partly

Are sufficient details of methods and analysis provided to allow replication by others?

Partly

Reviewer Expertise:

Molecular biology in aging and cancer

I confirm that I have read this submission and believe that I have an appropriate level of expertise to confirm that it is of an acceptable scientific standard.

# Reviewer report: Reviewer response for version 1

**DOI:** 10.5256/f1000research.171234.r395048

**Published:** 2025-07-22

**Authors:** Anom Bowolaksono

**Affiliations:** 1 Cellular and Molecular Mechanisms in Biological System (CEMBIOS) Research Group, Department of Biology, Faculty of Mathematics and Natural Sciences, Universitas Indonesia, Depok, Indonesia

This paper would be reviewed per section in points manner as would written below. There are some questions and critics those need to be cleared out in this paper

**Introduction**

- Here were said about average elimination half-life of the drug. Beforehand, it mentioned tramadol is converted into M1 and M2. So, which one is referred in the average elimination of drugs?
- Why does CYP2D6*5 alleles need to be studied? how does it significantly contribute to CYP2D6 activity levels?

**Methods**

- Standardization of VAS in this study is not really explained such as categorization criterions, patient symptoms, and valuation of VAS.
- Type of ANOVA model that used in this paper, for example Dunnet, Sidaks, Tukey, or else, is not clear.

**Result**

- It is hard to interpret from Long PCR results because legends each figures have different description. It always needs to recheck before seeing the results.
- As mentioned in **Methods**. Bar graph or table need are to be specified in related of statistical analysis beforehand of significancy conclusion.

**Discussion - Conclusion**

- There are some typos
- From this study, can it conclude the dose that might be works related to this study?

Is the work clearly and accurately presented and does it cite the current literature?

Yes

If applicable, is the statistical analysis and its interpretation appropriate?

Yes

Are all the source data underlying the results available to ensure full reproducibility?

Partly

Is the study design appropriate and is the work technically sound?

Partly

Are the conclusions drawn adequately supported by the results?

Partly

Are sufficient details of methods and analysis provided to allow replication by others?

Partly

Reviewer Expertise:

Molecular biology in aging and cancer

I confirm that I have read this submission and believe that I have an appropriate level of expertise to confirm that it is of an acceptable scientific standard, however I have significant reservations, as outlined above.

# Response

**Published:** 2025-08-21

**Authors:** Desriani Desriani

**Affiliations:** Genetic Engineering, Not Applicable, Bogor, Jawa Barat, Indonesia

This paper would be reviewed per section in points manner as would written below. There are some questions and critics those need to be cleared out in this paper

 >Thank you very much for your valuable input and comment.

**Introduction**

 Here were said about average elimination half-life of the drug. Beforehand, it mentioned tramadol is converted into M1 and M2. So, which one is referred in the average elimination of drugs?

- **Response: **Thank you very much for your comment. Please kindly find the explanation below. We have also included this explanation in the manuscript.

The two enantiomers that make up tramadol each contribute to its analgesic effects in a distinct way in cytochrome P450 system. The highly polymorphic enzyme CYP2D6 catalyzes the formation of M1 from O-desmethyltramadol. N-demethylation, however, was catalyzed by a different mechanism. As previously mentioned, the phenotypic variance of CYP2D6 is explained by its highly polymorphic character (mutation, deletion, and multiplication). Furthermore, depending on CYP2D6 phenotypes and other genetic factors, that the mean elimination half-life is around 6 hours, while the initial distribution half-life is 6 minutes. Three hours after delivery, O-Desmethyltramadol (M1), the main active metabolite of tramadol, reaches Cmax at 18–26% of the tramadol Cmax value. The average elimination half-life is roughly seven hours ^9,23^.

 Why does CYP2D6*5 alleles need to be studied? how does it significantly contribute to CYP2D6 activity levels?

- **Response: **Thank you very much for your comment. Please kindly find the explanation below. We have also included some of this explanation in the manuscript.

To date, prominent pharmacogenomics societies have developed clinical guidelines for at least 48 CYP2D6-substrate drugs. These guidelines contain therapeutic recommendations based on predicted CYP2D6 metaboliser phenotypes. The CYP2D6 pharmacogenomic guidelines use these metaboliser categories to provide clinicians with drug-specific recommendations, aiming to avoid prescribing ineffective or harmful doses to patients with a particular CYP2D6 metaboliser status. Phenotypic differences alter the capacity of enzymes to metabolize drugs.

 Deletion and multiplication of genes are important for phenotype prediction. Deletion of the entire CYP2D6 gene (*5) results in loss (in the homozygote) or reduction (in the heterozygote) of its protein production. ^3, 26, 27^ Furthermore, it is important to acknowledge that drug metabolism in the multiplication genotype increases the activity of the enzyme acitivity at a high rate as the functional increase. ^5, 22^ The UM phenotype may also be at risk of therapeutic failure or increased toxic side effects of metabolites. In this study showed that tramadol was significantly associated with the weight of postoperative patients receiving analgesia (p = 0.008). This indicated that administration of 100 mg of the drug resulted in optimal pain relief. As stated above, the typical dosage of tramadol administered is within the range of 50 to 100 milligrams. ^10^ The dosage for pediatric patients was 50 mg, whereas the adult dosage was 100 mg. Age and sex showed no significant correlations (p = 0.052 and 0.243, respectively). This could be interpreted as meaning that all genotypes in this activity individually metabolize the given tramadol is still possible. This also shows that both genotypes (heterozygote CYP2D6 *5 and the multiplication) are clinically functional.

**Methods**

 Standardization of VAS in this study is not really explained such as categorization criterions, patient symptoms, and valuation of VAS.

- **Response: **Thank you very much for your valuable comment. Please kindly find the explanation below. We have also included this explanation in the manuscript (in methods part).

The VAS is a subjective measure that depends on the pain tolerance of patients. However standarization of VAS scale was categorized as: 0 (no pain),  1-3 (signifying mild), 4-6 (middle), and 7-10 ( heavy pain). 

 We also provide additional information on the exclusion and inclusion criteria.

 All of the study's participants were patients who had undergone surgery at M. Djamil Padang Hospital. The inclusion criteria were: Patients with elective surgery at M. Djamil Hospital; proven Minangkabau ethnicity through family history; signed informed consent for blood sampling and pain assessment (VAS); and American Society of Anesthesiologists (ASA) criteria levels 1–2. The exclusion criteria were: Patients who refused to sign the informed consent form for sample collection; patients with ASA criteria levels ≥3 (patients with severe comorbidities e.g. renal failure or severe liver disease) that may interfere with tramadol metabolism, this also included patients who were taking other drugs that could significantly induce or inhibit CYP2D6 (e.g. fluoxetine, paroxetine and rifampicin).

 Type of ANOVA model that used in this paper, for example Dunnet, Sidaks, Tukey, or else, is not clear.

 Thank you for your valuable comments. The information about statistical analysis that was used ini this study was mentioned in the manuscript on “2.4 Statistical Analysis” section: “ *The demographic characteristics data were presented as the mean value ± standard deviation (SD). Subsequently, the data were analyzed with analysis of variance (ANOVA), followed by a chi-square test. The statistical analysis was conducted with the IBM SPSS software version 24, with p values less than 0.05 being related as statistically significant.”*

 Here it is the detailed explanation about statistical analysis using SPSS that has been used on the data consists of:

1. The significancies value for Visual Analogue Scale (VAS), age, sex, body weight, and molecular values, was firstly analyzed with Wild-typeity Test to evaluate the data distribution, after that ANOVA test was conducted and followed by Chi-square test. This was mentioned in 3. Results section, point 3.3  Patients demographics :

*“The sig value for VAS, age, sex, body weight, and molecular values <0.05 indicates that the data are not wild-typely distributed according to the Wild-typeity Test table. The demographic characteristics data presented as the mean value ± standard deviation (SD) showed no significant difference in VAS value, age, gender, and weight. The mean value range was 1.43-1.91, while the standard deviation ranged from 0.497 to 1.014 (detailed data were not shown). Further, the data was analyzed with analysis of variance (ANOVA), followed by a chi-square test.”*

1. The characteristic demographic of deletion, multiplication and wild-type polymorphism of CYP2D6 (age, sex, and weight as VAS measurement) was analyzed using Chi-square test to evaluate whether there is a correlation between the variables.
2. The correlation between the administration of tramadol and the Visual Analogue Scale (VAS) score (which indicates the pain ratin scale on patiens) was analyzed using ANOVA test (the data have been evaluated with Normality (Wild-typeity Test) and Homogenity tests, and the data was distributed normally and homogen).
3. The correlation between the administration of tramadol and the molecular examination was analyzed using ANOVA test (the data have been evaluated with Normality and Homogenity tests, and the data was distibuted normally and homogen).

**Result**

 It is hard to interpret from Long PCR results because legends each figures have different description. It always needs to recheck before seeing the results.

 >Thank you very much for your comments and input. We have updated the legend in all figures to be consistent. Please kindly see the updated manuscript.

 As mentioned in **Methods**. Bar graph or table need are to be specified in related of

 statistical analysis beforehand of significancy conclusion.

- **Response: **Thank you for your valuable comments. Please kindly find all the detailed statistical analyses that are reported in https://doi.org/10.6084/m9.figshare.27895956.v1, as mentioned in the data availability section of the manuscript.

**Discussion - Conclusion**

 There are some typos

- **Response: **Thank you very much for your comments and input. We have corrected the typos. Please kindly see the updated manuscript.

From this study, can it conclude the dose that might be works related to this study?

- **Response: **Thank you very much for your valuable comments and input.

Based on this study indicates that 100 mg of tramadol is an effective dose. In this study showed that tramadol was significantly associated with the weight of postoperative patients receiving analgesia (p = 0.008). This indicated that administration of 100 mg of the drug resulted in optimal pain relief. As stated above, the typical dosage of tramadol administered is within the range of 50 to 100 milligrams ^10^. The dosage for pediatric patients was 50 mg, whereas the adult dosage was 100 mg.   Age and sex showed no significant correlations (p = 0.052 and 0.243, respectively). This could be interpreted as meaning that all genotypes in this activity individually metabolize the given tramadol is still possible. This also shows that both genotypes ( heterozygote CYP2D6 *5 and the multiplication) are clinically functional.

# Reviewer report: Reviewer response for version 1

**DOI:** 10.5256/f1000research.171234.r391019

**Published:** 2025-07-01

**Authors:** Heri Setiawan

**Affiliations:** 1 Universitas Indonesia, Depok, Indonesia

This is an interesting study that describes the polymorphism of CYP2D6 in the Minangkabu ethic group. However, there are several aspects of this manuscript that should be revised:

 1. Please improve the narration:

 - "(wild-type, CYP2D6*5, and CYP2D6 multiplication)" Please explain shortly the metabolic capacity of each of these genotypes?

 - "This study had a retrospective clinical trial registry number NCT06642480." In my opinion, it is not necessary to include the trial registry number in the abstract.

 -"It is our first time using the clinical trial registry because the research already finished, so we applied for a retrospective registry clinical trial". This information may not be of concern to the reader

 -"Based on metabolic capacity, the phenotype of CYP2D6 can be divided into four parts: poor metabolizer (PM), intermediate metabolizer (IM), extensive metabolizer (EM), and ultra-metabolizer (UM)". Is there any phenotype that has a normal function of metabolism? Can the wild type in this study be considered a normal metabolizer?

 2. There is considerable explanation about the PCR method and its optimization, which has been published elsewhere. I think the scope of this study is the distribution of CYP26D and its clinical implications in the Minangkabau ethnicity. Therefore, please just show the best result of the PCR method.

 3. The images of the PCR product in gel electrophoresis are not clear, and the explanations are also inadequate.

 4. There is an inconsistency about the patient number. In the abstract and method, it is written that 63 patients were included. However, in Table 2, the total number of patients of both genders, all body weights, and ages is 65. Furthermore, the total number of patients with all VAS scores is 62.

 5. There is no explanation about the patient and its inclusion-exclusion criteria. It is important to control the confounding variables, such as disease severity, comorbidity, and the use of other medications.

 6. "This was evidenced in the present study, which showed a significant association between the drug and weight of postoperative patients (p = 0.008)." I think the association established in this analysis is between the class of body weight and genotype, which needs further explanation of its biological plausibility.

Is the work clearly and accurately presented and does it cite the current literature?

Partly

If applicable, is the statistical analysis and its interpretation appropriate?

Partly

Are all the source data underlying the results available to ensure full reproducibility?

Partly

Is the study design appropriate and is the work technically sound?

Partly

Are the conclusions drawn adequately supported by the results?

Partly

Are sufficient details of methods and analysis provided to allow replication by others?

Partly

Reviewer Expertise:

Pharmacology and toxicology

I confirm that I have read this submission and believe that I have an appropriate level of expertise to confirm that it is of an acceptable scientific standard, however I have significant reservations, as outlined above.

# Response

**Published:** 2025-08-21

**Authors:** Desriani Desriani

**Affiliations:** Genetic Engineering, Not Applicable, Bogor, Jawa Barat, Indonesia

This is an interesting study that describes the polymorphism of CYP2D6 in the Minangkabu ethic group. However, there are several aspects of this manuscript that should be revised:

- Thank you very much for your kind review and for considering our manuscript.

1. Please improve the narration:

 - "(wild-type, CYP2D6*5, and CYP2D6 multiplication)" Please explain shortly the metabolic capacity of each of these genotypes?

- Thank you very much for your kind input. We have revised the manuscript and added additional information. Please find the revised version below or in the first paragraph of the manuscript.…

According to the prevailing metabolic capacity, the CYP2D6 phenotype is classified into four distinct categories: poor metabolizer (PM), intermediate metabolizer (IM), extensive metabolizer (EM), and ultra-metabolizer (UM). ^3,4^ Arneth *et al* 2009 clasiffied EM as a characteristic of the normal population with two functional wild type alleles; PM usually results from the deletion or mutation of both alleles and is an autosomal recessive condition, IM those with one functional allele; Rapid metabolism UM is believed to be an autosomal dominant feature that results from either functional gene multiplication or duplication (>30%). ^3^ Further Darney *et al* 2021, reported that extensive metabolism as normal, and it is defined as having two functional wild-type alleles (i.e. *1, *2), or heterozygosity with one decreased-activity and one non-activity allele. PM is associated with CYP2D6 alleles with no activity both alleles (i.e., *3, *4, *5, *6, *7, *8, *11, *14, *15, *18, *19, *21, *29, *40). The IM phenotype has been associated with a decrease in CYP2D6 activity alleles, i.e. *9, *10, *17, *21, *36, *29, *41, *45, and *46, or heterozygosity for one decreased activity allele and one non-activity allele. The UM phenotype has been linked to at least one active CYP2D6 gene duplication. Patients  exhibiting the UM phenotype may face an elevated risk of therapeutic failure or an augmented propensity for adverse metabolic toxicities. ^4^

 - "This study had a retrospective clinical trial registry number NCT06642480." ****In my opinion, it is not necessary to include the trial registry number in the abstract. "It is our first time using the clinical trial registry because the research already finished, so we applied for a retrospective registry clinical trial". This information may not be of concern to the reader

- Thank you very much for your valuable comment. We input this information based on Editor F 1000 Research request.

-"Based on metabolic capacity, the phenotype of CYP2D6 can be divided into four parts: poor metabolizer (PM), intermediate metabolizer (IM), extensive metabolizer (EM), and ultra-metabolizer (UM)". ****Is there any phenotype that has a normal function of metabolism? Can the wild type in this study be considered a normal metabolizer?

- Thank you very much for your comment. As explained in the additional information in the first paragraph, extensive metabolisers are classified as the normal population. Further, in the case of our manuscript, we revised and consistently used the term 'wild type'. Please kindly see the updated manuscript.

2. There is considerable explanation about the PCR method and its optimization, which has been published elsewhere. I think the scope of this study is the distribution of CYP26D and its clinical implications in the Minangkabau ethnicity. Therefore, please just show the best result of the PCR method.

- Thank you very much for your kind comments and inputs. As we have no positive samples for the optimisation of long PCR for the wild-type, CYP2D6*5 and CYP2D6 multiplication targets, according to our opinion, besides reporting the distribution of CYP26D and its clinical implications in the Minangkabau ethnicity, we also should report how we modified and optimized the Johansson 1996 procedures for Long PCR.

The long PCR method developed by Johansson et al. (1996) 20 was modified in this study. The PCR products of wild-type CYP2D6, CYP2D6*5, and multiplication detection yielded 5 kb, 6 kb, and 10 kb, respectively. In contrast to Johansson et al. (1996), who used PerkinElmer's rTDNA and New England Biolabs' 0.09U Vent, the present study used Promega's GoTaqLong PCR Mastermix #M4021. GoTaq ® Long PCR Master Mix uses a combination of recombinant hot-start Taq DNA polymerase and recombinant proofreading DNA polymerase. The amplification conditions must be further optimized. In a preliminary study, the annealing temperature of 55°C-67°C was optimized using a three-step PCR (denaturation, annealing, and extension) for three DNA primers. No PCR product was detected (data not shown). Further, the annealing temperature across five different temperatures starting from 65 °C to 68°C was optimized using two-step PCR (denaturation, annealing also as an extension step) for each DNA primer. Among the five annealing temperatures tested, 68°C was the best temperature. One patient DNA sample with an 0 VAS scale, produced 6kb and 5kb for the amplified CYP2D6*5 DNA primer and wild-type CYP2D6 DNA primer, respectively, with no PCR product in all NTC. Using this 0 VAS scale patient DNA sample, amplification with a CYP2D6*5 DNA primer produced a 6 kb PCR product that consistently showed up at all annealing temperatures. The PCR product showed a specific amplification product at 68°C annealing temperature. In addition, amplification using a wild-type CYP2D6 DNA primer produced a 5 kb PCR product that consistently showed at all annealing temperatures, and specific PCR product were provide at 67.15°C and 68°C. Furthermore, DNA samples from other patients (second patient) that had 8 VAS scale showed only produced 5kb for the wild-type DNA primer at all annealing temperatures, and specific PCR product showed at 67.15°C and 68°C annealing temperature. The DNA primer used for the detection of CYP2D6 multiplication did not generate a PCR product for either sample ( Figure 1). This showed that the two samples used for optimization were CYP2D6*5 for the first sample (0 VAS scale) and the wild-type for the second sample (8 VAS scale). Specifically, CYP2D6*5 was identified as heterozygous CYP2D6*5 as the PCR product was produced using both CYP2D6*5 and wild-type DNA primers. According to Johansson et al., 20 heterozygous CYP2D6*5 led to PCR products with both CYP2D6*5 and wild-type DNA primers, whereas homozygous CYP2D6*5 resulted in no PCR product with wild-type DNA primers. Based on these results, there was an inconsistency between the VAS scale measurement and the genotype detection. This discrepancy was possible since VAS scale measurements are highly subjective and depend on patient pain tolerance.

 The optimal PCR setup was used to screen several samples. The screening results showed that one sample produced 10kb which was suspected to be a multiplication sample ( Figure 2). This sample was optimized for its optimal PCR setup, and the process was used to confirm and verify the results. Consistency was observed with a 10 kb PCR product as a single band across temperatures ranging from 63 to 68°C. The optimal PCR annealing temperature was determined to be 63.95, 65.2, and 67.15, indicating thick single-band DNA ( Figure 3). However, 68°C was selected because it produced a single band and was the most effective temperature for detecting both CYP2D6*5 and wild-type alleles. In addition, all the annealing optimization processes showed clear and clean NTC. The remaining samples were screened using a modified method.

 3. The images of the PCR product in gel electrophoresis are not clear, and the explanations are also inadequate.

- Thank you very much for your comment and we regret to inform you that, at that moment, our gel doc DNA tray was in a crack condition. We believe that, despite the crack situation, our DNA target was clear and visible. We also indicated the DNA target with an arrow, as shown in the figure in the text. Further We add additional explanation regarding the PCR in order have adequate explanation (highlighted with yellow). Please kindly find below or in the manuscript

This study aimed to investigate CYP2D6*5 and CYP2D6 multiplication in post-operative Minangkabau ethnicity patients receiving tramadol analgesia and further investigate the efficacy of tramadol in these patients. To achieve this, the Long PCR method developed by Johansson et al., 20 was modified. The Long PCR, reported by Johansson et al 1996, was successfully modified with the GoTaq Long PCR master mix from Promega #M4021, with two-step PCR at 68°C annealing and extension PCR amplification temperatures.  It is important to optimise the annealing temperature, particularly when synthesising long PCR products or using total genomic DNA as the PCR substrate.  An annealing temperature that is too high will reduced the PCR product yielded while an annealing temperature that is too low will produce an unspecific PCR product. PCR annealing temperature optimization was one of the key successes for developing PCR methods. ^21^. GoTaq ® Long PCR Master Mix uses a combination of recombinant hot-start Taq DNA polymerase and recombinant proofreading DNA polymerase. Both enzymes in the GoTaq Long PCR master mix from Promega #M4021 were required to amplify long targets. Taq DNA Polymerase alone cannot correct nucleotide mismatches resulting from misincorporation and is therefore ineffective in amplifying segments longer than 3-5 kb. Products are truncated if nucleotides are misincorporated; further, they cannot be extended in subsequent cycles. Long amplicons can be amplified with a high yield by adding a small amount of proofreading enzyme to fix mismatches and allow extension. To ensure accurate PCR, the size of the product was monitored. The sizes of the wild-type, CYP2D6*5, and CYP2D6 multiplication PCR products were 5kb, 6kb, and 10 kb, respectively. The wild-type genotype PCR product was used as an control reaction. Furthermore, heterozygous CYP2D6*5 produced a DNA PCR product in a tube containing CYP2D6*5 and wild-type DNA primers. In contrast, homozygous CYP2D6*5 produced no PCR products in the tube containing wild-type DNA primers. Although the real-time method offers a simpler and faster alternative to long PCR, it requires costly real-time PCR machines, and pyrosequencing is also costly. The modified Long PCR method was used to generate a distribution report of the Minangkabau ethnicity genotypes.

 4. There is an inconsistency about the patient number. In the abstract and method, it is written that 63 patients were included. However, in Table 2, the total number of patients of both genders, all body weights, and ages is 65. Furthermore, the total number of patients with all VAS scores is 62.

- Thank you very much for your kind correction. This study included a total of 65 patients. Among the patients, 62 received tramadol analgesia and 63 were tested for genetics, while the other two samples did not have detailed information. Please kindly see detailed information in https://doi.org/10.6084/m9.figshare.26870995, as mentioned in the data availability section of the manuscript.

5. There is no explanation about the patient and its inclusion-exclusion criteria. It is important to control the confounding variables, such as disease severity, comorbidity, and the use of other medications.

 Thank you very much for your kind input. Please kindly find our manuscript that we have added and completed the inclusion and exclusion criteria, In the methods section.

 ..All of the study's participants were patients who had undergone surgery at M. Djamil Padang Hospital. The inclusion criteria were: Patients with elective surgery at M. Djamil Hospital; proven Minangkabau ethnicity through family history; signed informed consent for blood sampling and pain assessment (VAS); and American Society of Anesthesiologists (ASA) criteria levels 1–2. The exclusion criteria were: Patients who refused to sign the informed consent form for sample collection; patients with ASA criteria levels ≥3 (patients with severe comorbidities e.g. renal failure or severe liver disease) that may interfere with tramadol metabolism, this also included patients who were taking other drugs that could significantly induce or inhibit CYP2D6 (e.g. fluoxetine, paroxetine and rifampicin)…

 6. "This was evidenced in the present study, which showed a significant association between the drug and weight of postoperative patients (p = 0.008)." I think the association established in this analysis is between the class of body weight and genotype, which needs further explanation of its biological plausibility.

- Thank you very much for your kind comment. Please find the additional explanation below.

The two enantiomers that make up tramadol each contribute to its analgesic effects in a distinct way in cytochrome P450 system. The highly polymorphic enzyme CYP2D6 catalyzes the formation of M1 from O-desmethyltramadol. N-demethylation, however, was catalyzed by a different mechanism. As previously mentioned, the phenotypic variance of CYP2D6 is explained by its highly polymorphic character (mutation, deletion, and multiplication). Furthermore, depending on CYP2D6 phenotypes and other genetic factors, that the mean elimination half-life is around 6 hours, while the initial distribution half-life is 6 minutes. Three hours after delivery, O-Desmethyltramadol (M1), the main active metabolite of tramadol, reaches Cmax at 18–26% of the tramadol Cmax value. The average elimination half-life is roughly seven hours. ^9 , 25^ Deletion and multiplication of genes are important for phenotype prediction. Deletion of the entire CYP2D6 gene (*5) results in loss (in the homozygote) or reduction (in the heterozygote) of its protein production. ^3, 26, 27^ Furthermore, it is important to acknowledge that drug metabolism in the multiplication genotype increases the activity of the enzyme acitivity at a high rate as the functional increase. ^5, 22^ The UM phenotype may also be at risk of therapeutic failure or increased toxic side effects of metabolites. In this study showed that tramadol was significantly associated with the weight of postoperative patients receiving analgesia (p = 0.008). This indicated that administration of 100 mg of the drug resulted in optimal pain relief. As stated above, the typical dosage of tramadol administered is within the range of 50 to 100 milligrams. ^10^ The dosage for pediatric patients was 50 mg, whereas the adult dosage was 100 mg. Age and sex showed no significant correlations (p = 0.052 and 0.243, respectively). This could be interpreted as meaning that all genotypes in this activity individually metabolize the given tramadol is still possible. This also shows that both genotypes (heterozygote CYP2D6 *5 and the multiplication) are clinically functional.

 The potency of tramadol analgesia is approximately 10% that of morphine when administered intravenously. Tramadol has also been reported to be comparable to morphine. ^28, 29^

 Tramadol is an efficient and well-tolerated medication for the treatment of chronic pain, whether of malignant or non-malignant origin, especially neuropathic pain. It can also be used to relieve pain related to trauma, renal or biliary colic, and labor. When taken as a non-opioid analgesic, the analgesic effect of the drug can be enhanced. 28 Furthermore, tramadol administration is ideally based on body weight. According to Sidiq et al. (2015), 2 mg/kg body weight of tramadol hydrochloride is an optimal dose for postoperative analgesia when administered epidurally in urological surgery, with no significant increase in side effects. 30 Pang et al. (2005) reported that the recommended intraoperative dose of tramadol is 2.5 mg/kg body weight for effective postoperative analgesia with minimal sedation, when considering the efficacy and side-effect profile. ^29^

 Patients without CYP2D6 activity may not be able to metabolize drugs. Ultra-rapid metabolizers may also experience treatment failure owing to the rapid metabolism of active drugs, resulting in drug levels below the therapeutic range. 5 The data obtained specifically in the multiplication part were not in line with the presented statement. This discrepancy may be attributed to the absence of a subsequent follow-up. To enhance the quality of future data, it is essential to categorize the type of operation, collect a larger number of samples, and conduct longer follow-up periods, and also estimate the pharmacokinetic parameter of tramadol in plasma. Tramadol's enantiomers, their primary phase I metabolites, and epinephrin were pharmacokinetic parameter of tramadol in plasma in order to evaluate the CYP2D6 phenotype. ^23^ Also following the recommendation of the consensus on translating CYP2D6 genotype to metabolizer phenotype. ^24^
